# Load monitoring on Pilates training: a study protocol for a randomized clinical trial

**DOI:** 10.1186/s13063-019-3684-x

**Published:** 2019-10-17

**Authors:** Allysiê Priscilla de Souza Cavina, Eduardo Pizzo Junior, Aryane Flauzino Machado, Taíse Mendes Biral, Carlos Marcelo Pastre, Franciele Marques Vanderlei

**Affiliations:** 1 Postgraduate Program in Physiotherapy, Universidade Estadual Paulista (FCT/UNESP), Presidente Prudente, SP Brazil; 2 Faculdade de Ciências e Tecnologia – FCT/UNESP, Departamento de Fisioterapia, Rua Roberto Simonsen, 305 – Cidade Universitária, Presidente Prudente, SP 19060-900 Brazil; 3 Graduate in Physiotherapy, Universidade Estadual Paulista (FCT/UNESP), Presidente Prudente, SP Brazil; 4 Department of Physiotherapy, Universidade Estadual Paulista (FCT/UNESP), Presidente Prudente, SP Brazil

**Keywords:** Exercise movement techniques, Exercise, Monitoring, Physiologic, Heart rate, Autonomic nervous system

## Abstract

**Background:**

Currently there are campaigns to raise the awareness of the need to practice physical exercise with several objectives, mainly as a preventive measure. The Pilates method is a form of therapeutic exercise for maintaining and improving health. However, despite being popular, there is still no scientific evidence on the standardization and progression of the method. Therefore, the purpose of this study was to develop a protocol to monitor the progression of daily Pilates loads between the basic, intermediate, and advanced levels, as well as to analyze the effects of the method on psychometric, cardiorespiratory, and autonomic measures.

**Methods/design:**

In total, 54 healthy men underwent 36 sessions of Pilates mat work. Before each training session, cardiorespiratory measures, pain (visual analogue scale), and a psychometric questionnaire were collected. Heart rate (HR), subjective perception of effort (SPE), and RR intervals were measured during the sessions and used later in the analysis of the progression of training load by monitoring the internal training load and heart rate variability. At the end of the sessions, cardiorespiratory measures, the visual analogue scale, and the psychometric questionnaire were measured again. After 15 min of rest, the final HR measurement was made and the participants noted the effort on the SPE scale. The psychometric, cardiorespiratory, and autonomic measures were evaluated before and after each of the 36 training sessions.

**Discussion:**

This is a parallel randomized clinical trial of standardized Pilates training, with the aim of estimating training loads and measuring the efficacy of Pilates through clinical, cardiorespiratory, and autonomic outcomes. The protocol can easily be reproduced and could be used to support professionals in prescribing the method.

**Trial registration:**

ClinicalTrials.gov, NCT03232866. Registered on 28 July 2017.

**Electronic supplementary material:**

The online version of this article (10.1186/s13063-019-3684-x) contains supplementary material, which is available to authorized users.

## Background

The Pilates method is an alternative therapeutic exercise for protecting against risk factors and promoting health [1]. However, only one literature review investigates the Pilates method [2]. The methodological quality is low, which demonstrates the lack of standardization of the method and the load progression as a form of physical training [2]. Thus, for the safe prescription of resistance exercises, such as the Pilates method, physiological variables, such as heart rate (HR) [3] and cardiac autonomic modulation [4, 5], and subjective variables, such as subjective perception of effort (SPE) [6], need to be monitored.

Thus, there is a need to identify simple and low-cost methods for controlling the intensity of exercises, due to the adaptations made by the psychobiological system through training, such as improvements in exercise tolerance and physical performance [7]. It would be beneficial to use SPE together with HR to quantify the training intensity of the Pilates method, as well as to use cardiac autonomic modulation to assess the oscillations of the cardiac autonomic nervous system during training, since heart rate variability (HRV) is considered an early and sensitive indicator of health impairment. HRV indicates the adaptability of cardiac autonomic control to a stimulus [8, 9].

Although the Pilates method is becoming more widespread and has shown promising clinical and functional outcomes in rehabilitation [10, 11], quality clinical trials are needed to analyze the intensity of the training. The results of such trials may offer greater support for tools that can assist in the prescription and load progression of Pilates practice, while demonstrating the effects of the method on the cardiovascular system and autonomic control.

Therefore, the objective of this protocol is to monitor the daily load progression from the basic and intermediate levels to the advanced level by measuring HR, SPE, and HRV for 12 weeks, as well as to analyze the effect of the method on psychometric, cardiorespiratory, and autonomic measures. It is hypothesized that the protocol proposed by the study can be prescribed clinically, proving that SPE, HR, and HRV are useful tools for monitoring the progression of Pilates training loads. Furthermore, SPE is expected to have a good correlation with HR and clinical relevance, thus giving practitioners two simple low-cost monitoring tools. In addition, it is hypothesized that the psychometric, cardiorespiratory, and cardiac autonomic modulation measures will have improved after 12 weeks of training.

## Methods/design

### Study design

A parallel randomized clinical trial is being conducted at the Center for Studies and Assistance in Physiotherapy and Rehabilitation of the Universidade Estadual Paulista (FCT/UNESP), Presidente Prudente, São Paulo, Brazil. The trial was registered at ClinicalTrials.gov (NCT03232866) and approved by the research ethics committee of FCT/UNESP (protocol 061942/2017). The study protocol follows the SPIRIT 2013 checklist (Standard Protocol Items: Recommendations for International Trials) [12] (Additional file 3) and TIDieR (Template for Intervention Description and Replication) [13], indicating that information about and the quality of the reports of the interventions are well described [14]. Prior to participation, the volunteers received oral and written instructions and signed a consent form agreeing to participate in the study. All personal data will remain confidential. Note that, although very unlikely, participants who suffer any kind of injury as a result of participation will receive a free medical evaluation and physiotherapy treatment.

**Fig. 1:**
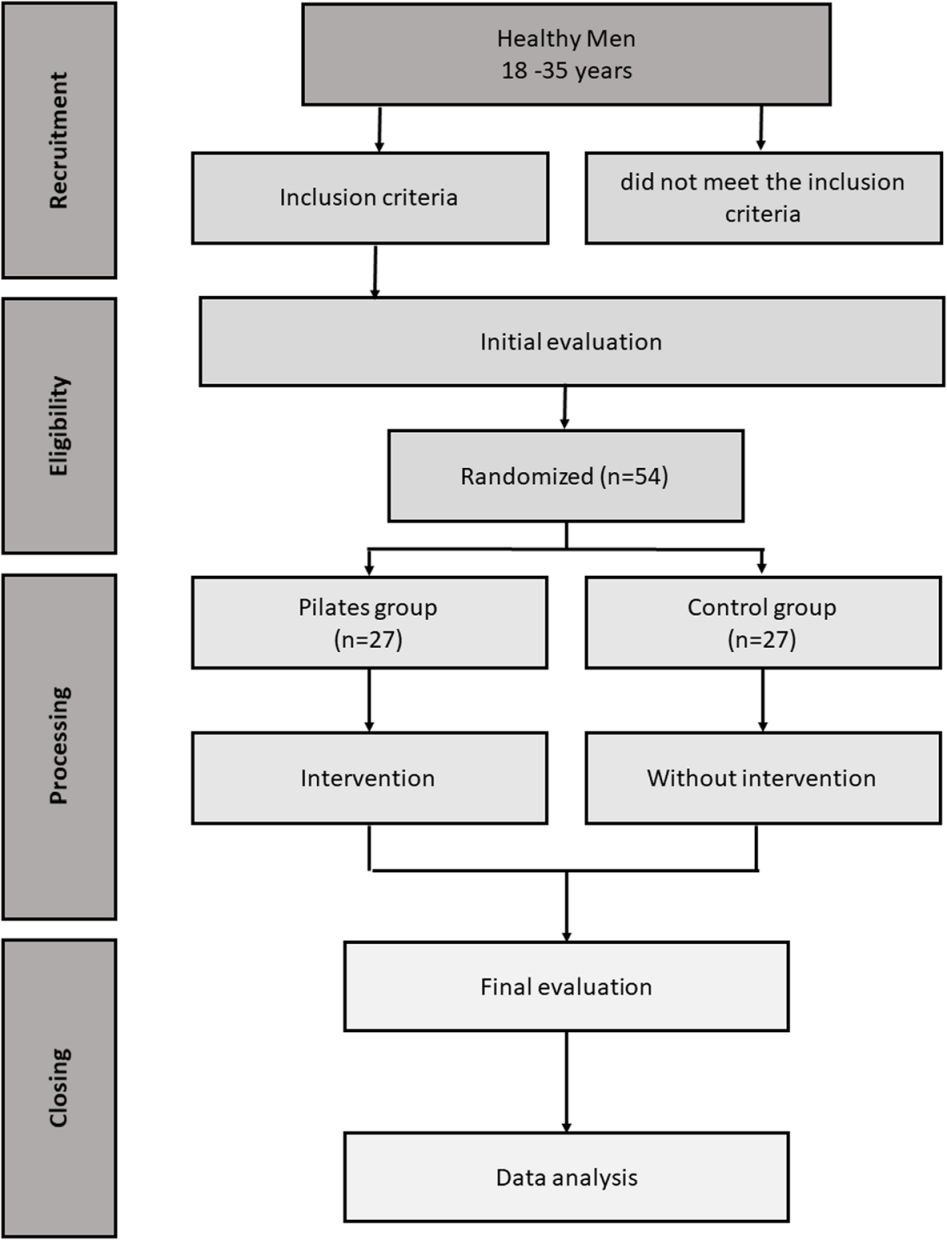
Flowchart of the study

Participants were divided into two groups: the Pilates group and the control group. Participants in the Pilates group underwent 12 weeks of Pilates exercises for three times a week, for a total of 36 sessions. Each session lasted for approximately 60 min. During this training period, participants were required to pass through the three levels of the Pilates method: basic, intermediate, and advanced. The control group were asked to maintain their current level of daily activities without taking any type of exercise training. A flowchart for the study and group composition are shown in Fig. 1.

### Participants

A total of 54 healthy male volunteers were recruited from the local university (UNESP) through pamphlets, online media, personal invitations, telephone calls, and SMS messages. These procedures were recommended by Treweek et al. [15] as strategies to improve participant recruitment.

The inclusion criteria were: male; 18 to 35 years of age; healthy (self-report); not having practiced Pilates prior to the study; not a smoker or an alcoholic; not presenting with a metabolic, cardiac, or endocrine disorder; with no arrhythmia; not using drugs that influence the autonomic modulation of the heart; and without physical limitations such as musculoskeletal, inflammatory, or neurological diseases that would prevent them from doing Pilates.

Individuals who suffered from a musculoskeletal injury during training, those who attended less than 85% of training sessions, those who failed to progress between the three levels of the Pilates method, those who were unable to respond adequately to subjective scales, and those with errors in the captured RR intervals were excluded from the study. Participants were instructed to maintain their daily dietary routine, to abstain from taking anti-inflammatory medications and analgesics, and not to perform any other type of exercise during the intervention period. These requests were reinforced during the training period and monitored by self-report [16].

### Randomization

Prior to randomization, baseline data were collected from participants who meet the eligibility criteria and had signed the informed consent form. To maintain the blinding, randomization by group (Pilates group or control group) was performed by another researcher not involved in the recruitment using a computer-generated randomization schedule.

### Details of procedures

#### Study outline

The evaluations were carried out at FCT/UNESP, in a room with controlled temperature and humidity, at the same time of day. For the initial evaluation, after completing the personal information, the participants completed a psychometric questionnaire [17]. Once the anthropometric metrics had been collected for each participant, their body mass index was calculated.

In addition, systolic and diastolic blood pressure, HR, respiratory rate, and oxygen saturation were measured. Then, the participants were sent to a quiet room where a cardiofrequency meter (V800, Polar Electro Oy, Kempele, Finland) recorded their HR beat to beat for 20 min for later analysis of HRV.

**Fig. 2:**
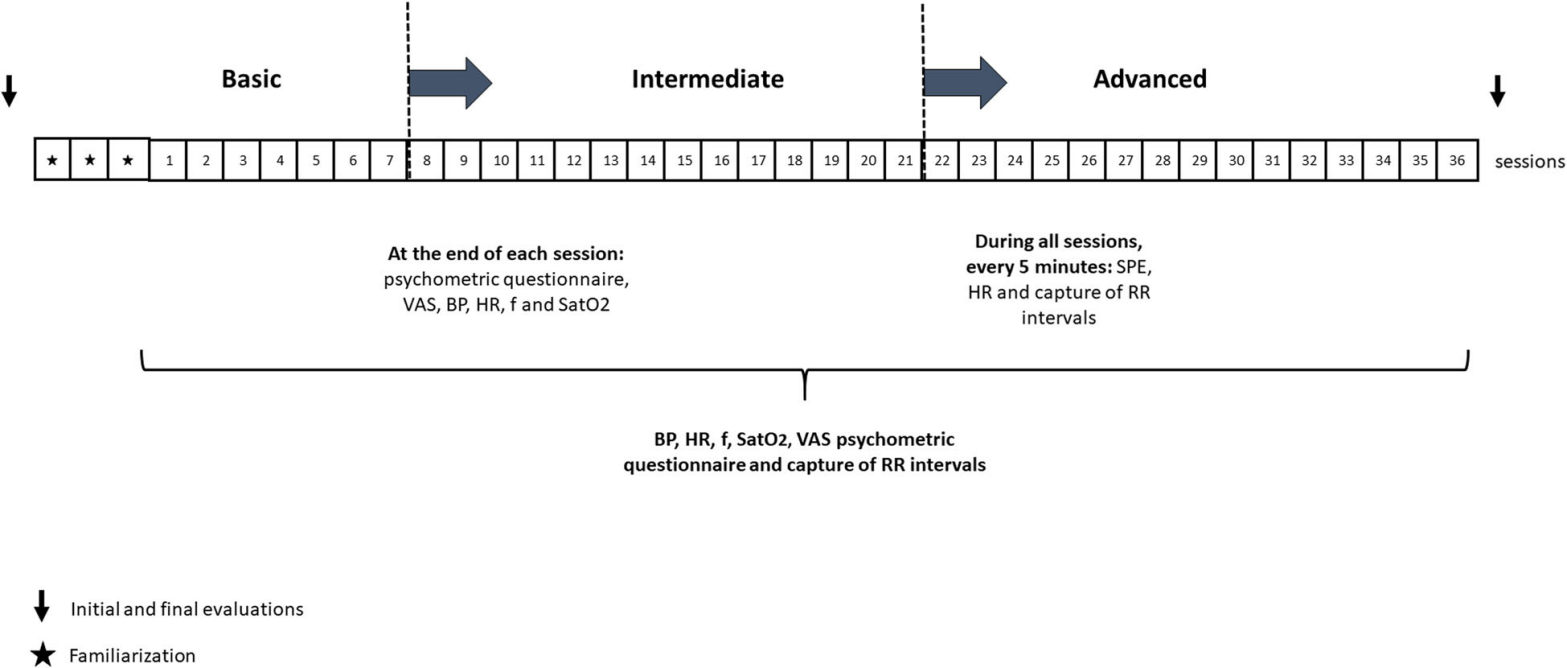
Study outline. VAS visual analog scale BP blood pressure, HR heart rate, *f* respiratory rate, SatO_2_ oxygen saturation, SPE subjective perception of effort

The Pilates training began after a week with three familiarization sessions. At the start of each training session, cardiorespiratory measures (systolic blood pressure, diastolic blood pressure, respiratory rate, HR, and oxygen saturation) and pain (visual analogue scale) were measured, and a psychometric questionnaire was completed, as the basal control. To measure the progression of load, HR, SPE, and vagal indices of HRV were used. These metrics were collected during the exercises. HR was measured and recorded on the SPE scale every 5 minutes, and the RR intervals were recorded during the whole session. At the end of each session, the cardiorespiratory metrics and pain (visual analogue scale) were measured, and the psychometric questionnaire was completed. After 15 minutes of rest, the volunteers underwent the final HR measurements and completed the SPE scale again. The sessions are shown in Fig. 2.

At the end of the 36 Pilates training sessions, the final evaluation was carried out. This included cardiorespiratory measures, the psychometric questionnaire, and HRV. The control group underwent only the initial and final evaluations, like the Pilates group.

### Pilates exercises

The exercises are shown in Additional file 1. The exercise sequences were divided in different ways to make the training more varied and to avoid dropouts (Additional file 2). The exercise schedule was produced by us based on an extensive bibliographical survey of Pilates exercises and their progression. We considered the degree of difficulty of each exercise as well as the dependence between volume and intensity.

### Primary outcome

Quantification of the internal training load was evaluated in each training session with the three monitoring tools: SPE [6], HR [18], and HRV [18]. For the monitoring to be considered successful, two of the three monitoring tools must yield significant results. In addition, the correlation between SPE and HR should be moderate to high so that they can be used in clinical practice as low cost and easily applicable tools.

The SPE consists of a scale proposed by Borg where the participants choose the descriptor and number that best represents their psychophysiological stress in the training session [6]. It is a scale from 6 to 20 points, where 6 means “no effort” and 20 means “maximum effort.” To avoid biased responses, the question was standardized as: “From 6 to 20 points, how do you rate your perception of effort now?” All responses were recorded in individual records. The participants had previously been made familiar with the scale [6].

Prior to the sessions, participants were told how to estimate their SPE. It was explained to them that during the exercises it was necessary to perceive how much effort they were using so that every 5 minutes during a session they could estimate the SPE according to their individual perception for how exhausting the exercises were.

In addition, 15 min after the end of each session, the participants estimated the SPE again [6]. This final SPE was multiplied by the duration of the session in minutes, and the product represents the internal load in arbitrary units. The intensity of the session was classified into three zones according to the Borg scale used by Moreira et al. [19].

Quantification of the internal load training using the HR was performed using the TRIMP method. This evaluates the volume and intensity of the session through specific scores in each training zone, as proposed by Edwards [18, 20].

In addition to these methods for monitoring the training load, HRV was analyzed. The weekly mean of rMSSD (rMSSDmean) expressed in milliseconds and the weekly intra-individual coefficient of variation of rMSSD (rMSSDcv) expressed as a percentage will be used [21, 22]. These indices will be transformed to logarithms to mitigate outliers and simplify the analysis.

### Secondary outcomes

The HRV will be calculated from the series of RR intervals captured by the cardiofrequency monitor using linear methods. It will be analyzed in the time and frequency domains. A Poincaré plot will be used. All HRV indices will be calculated by Kubios HRV software version 3.1.0.

The RR interval series was recorded before and after the 12 weeks of training. There were 1000 RR intervals in each data set. In addition, daily tracking of each session will be analyzed. Stretches of 256 consecutive RR intervals were recorded every 5 minutes. In both analyses, the most stable stretches were used to avoid any bias in the analysis and data interpretation [5]. The time series of RR intervals will undergo moderate digital filtering using Kubios, supplemented by manual filtering to eliminate premature ectopic beats and artifacts. Only series with more than 95% of sinus beats will be included in the final analysis [23].

The time domain (rMSSD and standard deviation of all RR intervals) [24], frequency (low and high) [23, 25, 26], and Poincaré plot (standard deviation of data from *x* = *y* and standard deviation of data from the line orthogonal to *x* = *y* that passes through the mean value of the data) [23, 27, 28] HRV indices will be used for the analyses before and after the 12 weeks of training. Only the time domain indices and the Poincaré plot will be used for daily tracking.

HR [23], respiratory rate measurements [29], and oxygen saturation [30] were captured. Blood pressure [31] was measured. A subjective pain assessment was obtained through a visual analog pain scale [32, 33]. Volunteers were asked to fill out a psychometric questionnaire [17] before and after the training period, as well as before all training sessions.

All these outcomes were collected in the initial evaluation and in the final evaluation after the 12 weeks of training, in addition to before and after each Pilates session. Table 1 lists outcome collection time points.

**Table 1:** Time points for outcomes

Outcomes			Time point				
			Initial assessment	Final evaluation	During the sessions		
					Before and at the end	Every 5 min	15 min after
Primary	Load monitoring	Borg			X	X	X
		HR			X	X	X
		rMSSDmean			X	X	
		rMSSDcv			X	X	
Secondary	ANS	HRV	X	X	X	X	
	Cardiorespiratory metrics	BP	X	X	X		
		HR	X	X	X		
		*f*	X	X	X		
		SatO_2_	X	X	X		
	Pain	VAS	X	X	X		
	SPE	Borg	X	X			

*SPE* subjective perception of effort, *HR* heart rate, *ANS* autonomic nervous system, *HRV* heart rate variability, *BP* blood pressure, *VAS* visual analog scale, *f* respiratory rate, *SatO*_*2*_ oxygen saturation, *rMSSD* parasympathetic modulation index, *rMSSDmean* weekly mean of rMSSD expressed in milliseconds, *rMSSDcv* weekly intra-individual coefficient of variation of rMSSD

### Masking and blinding

Baseline outcomes were collected prior to randomization. The final evaluation was conducted by trained evaluators blinded to group allocation. In addition, data were collected throughout the Pilates training period by independent evaluators. The study statistician will also be blinded. In this study, it was not possible to blind either the participants or the therapist who administered the Pilates sessions.

### Sample size calculation

The sample calculation was carried out based on Barbosa et al*.* [34] with the rMSSD index. The clinically relevant difference was 19,02 ms and the standard deviation 23,94 ms. For a two-tailed hypothesis test, the significance level for the sample calculation was 5% and the power of the test was 80%. The sample size was calculated to be 25 per group. To account for withdrawals, the sample size was increased by 10% to give 27 volunteers per group.

### Statistical analysis

Descriptive statistics will be used, and the results presented as means, errors, standard deviations, percentages, and absolute numbers. The normality of the data will be evaluated through a Shapiro–Wilk test. In the comparison of the population profile and the cardiorespiratory, autonomic, and psychometric measures in the initial and final evaluations, the paired *t* test will be used for normal data or the Wilcoxon test for non-normal data. In the comparison between the groups, the unpaired *t* test or Mann–Whitney test will be used depending on the normality of the data, with the significance level set at *p* < 0.05. Differences based on magnitudes (Δ = final value – initial value) will also be calculated [35, 36] to verify the differences between the initial and final time points.

To analyze weekly differences in training loads using the SPE, HR, and HRV indices (ln rMSSDweekly and ln rMSSDcv) over the 12-week study duration, magnitude-based inferences will be calculated [36].

For ln rMSSDweekly, the smallest worthwhile change was set at 3% [37]. For SPE, HR, and ln rMSSDcv, the smallest worthwhile change is defined as 0.2 × between-subject standard deviation. The quantitative chances to find differences in the variables to be tested will be assessed qualitatively as follows: <1% almost certainly not, 1–5% very unlikely, 5–25% unlikely, 25–75% possible, 75–95% likely, 95–99% very likely, and >99% certain. If the chances of having higher and lower results are both >5%, the true difference was assessed as nuclear [35]. The magnitude of the effect size will be qualitatively interpreted using the following thresholds: <0.2 trivial, 0.2–0.6 small, 0.6–1.2 moderate, 1.2–2.0 large, 2.0–4.0 very large, and >4.0 nearly perfect [35].

To determine the correlations (dose–response) between ln rMSSD, HR, and SPE, Pearson’s or Spearman’s correlation will be used according to the normality of the data. The threshold used to evaluate the correlations quantitatively will be based on Hopkins [35] using the following criteria: <0.1 trivial, 0.1–0.3 small, 0.3–0.5 moderate, 0.5–0.7 large, 0.7–0.9 very large, and >0.9 almost perfect.

The cutoff points for load increments with the HRV indices and SPE were obtained from the receiver operating characteristic curve. The sensitivity, specificity, positive predictive value, and negative predictive value for each level of the Pilates method will also be calculated. The area under the curve will be considered significant when values ≥0.65 are obtained [38]. In addition, a comparison will be made between the receiver operating characteristic curves of each HRV index to detect the most representative for determining the internal training load of the Pilates method.

The statistical analysis will be carried out with the *Statistical Package for the Social Sciences*, version 15.0 (SPSS Inc., Chicago, IL) and *MedCalc Software bvba*, version 14.10.2 (Oostende, Belgium). All data will be entered into the software twice. All participants included in the initial assessment will be considered under the intention-to-treat approach in which the worst outcome obtained by the study group will be selected to ensure the power of the data analysis.

## Discussion

Previous systematic reviews have found a beneficial effect of Pilates on risk factors for various diseases, as well as positive responses in outcomes such as strength, flexibility, low back pain, and quality of life, among others [2, 39, 40]. However, the low methodological quality of previous studies and the lack of load monitoring of the exercises do not allow definitive conclusions to be drawn about the effect of the Pilates method. Moreover, there have been few studies investigating the effects of Pilates on the cardiac autonomic nervous system.

Methodological characteristics commonly found in studies published in the literature include lack of concealed allocation, no intention-to-treat analysis, unblinded assessors, lack of standardization of training on biological principles, and low familiarization of participants with the method. This study is a parallel randomized clinical trial to measure the effectiveness of the Pilates method on the cardiac autonomic nervous system and, mainly, to analyze methods of monitoring training loads.

The best measures for quantifying the internal load are SPE and HR, because they are cheap and simple to use. They can be measured by any healthcare professional. HRV is also important as an early and sensitive indicator of health impairment; however, correct analytical use of this tool requires detailed knowledge.

The training protocol described in this study was based on the most recent literature. A strength of the study is the parallel randomized clinical trial design with an intention-to-treat analysis. Moreover, the protocol is easy to replicate. However, a limitation is the inability to blind participants to their allocation.

This study may contribute by providing further information on the real autonomic benefits provided by this type of physical exercise and can be used to guide practitioners in prescribing the method, as well as offering more support to practitioners.

Note that this study uses the checklist of items for protocol studies to minimize bias. It was prospectively registered. The outcomes will be disseminated through publications in scientific journals and presentations at area congresses.

### Trial status

The registration number is NCT03232866. The study start date was 1 September 2018. The primary completion date was May 2019. Patient recruitment is completed. The data were collected in June 2019. Altogether, 58 volunteers were assessed for eligibility, of whom four were excluded, so that 54 participants were randomized. All participants are attending ongoing study groups. The study completion date is December 2019.

## Additional files

Additional file 1: Pilates exercises. (TIF 5581 kb)

Additional file 2: Pilates exercise sequences. (DOCX 17 kb)

Additional file 3: SPIRIT 2013 Checklist: Recommended items to address in a clinical trial protocol and related documents. (DOC 125 kb)

## Data Availability

Not applicable.

## Abbreviations

ANS: Autonomic nervous system
FCT/UNESP: Center for Studies and Assistance in Physiotherapy and Rehabilitation of the Universidade Estadual Paulista
HR: Heart rate
HRV: Heart hate variability
rMSSD: Root mean-square of successive differences of adjacent RR intervals
rMSSDcv: Weekly intra-individual coefficient of variation of rMSSD
rMSSDmean: Weekly mean of rMSSD expressed in milliseconds
SatO_2_: Oxygen saturation
SMS: Short Message Service
SPE: Subjective perception of effort
VAS: Visual analog scale
