# Supplementary material for: Load monitoring on Pilates training: a study protocol for a randomized clinical trial
Source: Trials. 2019 Oct 17;20:597. doi: 10.1186/s13063-019-3684-x (PMC6798512; doi:10.1186/s13063-019-3684-x)
Supplement: Supplementary file 2 — Pilates exercise sequences. (DOCX 17 kb) [file 13063_2019_3684_MOESM2_ESM.docx]

# Additional file 2. Pilates method exercise sequences

## Session A

| **BASIC**  **Session 1-7** | **INTERMEDIATE**  **Session 8-21** | **ADVANCED**  **Session 22-36** |
| --- | --- | --- |
| **Exercises (15 repetitions)** | **Exercises(12 repetitions)** | **Exercises(10 repetitions)** |
| Preparation 1 to The Hundred  Roll Up modified  Preparation to Single Leg Circles  Single leg stretch  Double leg stretchmodified  Obliques  Spine stretch forward  Saw  Side leg lift/ lateral flexion  Sidekicks/small circles  Sidekicks/up down  Preparation to Shoulder Bridge  Preparation to Teaser  Preparation to Swimming  Obliques Roll Back  Preparation to Side Bend  Preparation Push-ups | Preparation 3 to The Hundred  Roll Up  Roll over  Single Leg Circles  Single leg Stretch  Double leg stretch  Crisscross  Open leg Rocker  Saw modified  Neck Pull  Preparation to Jack knife  Shoulder Bridge  Sidekicks/small circles  Sidekicks/inner-thigh lifts  Teaser I  Preparation to Hip Circles  Swimming  Preparation to Leg Pull Front  Preparation to The leg pull-up  Kneeling  Preparation to Side Bend  Preparation to Push-ups | The Hundred  Single straight leg stretch modified  Double straight leg stretch modified  Corkscrew modified  Scissors  Shoulder Bridge modified  Spine Twist  The Jackknife  Sidekicks/up down modified  Sidekicks/front back modified  inner-thigh lifts modified  Sidekicks/Bicycle  Teaser II  Hip Circle  Leg Pull Front  The leg pull-up  Side Bend  The Boomerang  Rocking  Rowing 1 |
|  |  |  |
|  |  |  |

## Session B

| **BASIC**  **Session 1-7** | **INTERMEDIATE**  **Session 8-21** | **ADVANCED**  **Session 22-36** |
| --- | --- | --- |
| **Exercises (15 repetitions)** | **Exercises (12 repetitions)** | **Exercises (10 repetitions)** |
| Preparation 2 to The Hundred  Roll Up  Preparation to Single Leg Circles  Single leg stretch modified  Double leg stretch  Obliques  Spine stretch forward  Saw  Obliques Roll Back  Preparation to  Shoulder Bridge  Sidekicks/small circles  Sidekicks/up down  Side leg lift/ lateral flexion  Preparation to Teaser  Preparation to Swimming  Preparation to Side Bend  PreparationtoPush-ups | Preparation 3 to The Hundred  Roll up  Roll over  Single straight leg stretch  Double straight leg stretch  Double leg stretch  Spine Stretch forward  Corkscrew  Saw  Neck Pull  Shoulder Bridge  Preparation to Jack knife  Sidekicks/small circles  Sidekicks/inner-thigh lift  Teaser I  Preparation to Hip Circles  Swimming  Preparation to Leg Pull Front  Preparation to The leg pull-up  Keeling  Preparation to Side Bend | The Hundred  Single straight leg stretch modified  Double straight leg stretch modified  Corkscrewmodified  The Bicycle  Shoulder Bridge modified  Spine Twist  Jack knife  Sidekicks/small circles modified  Sidekicks/inner-thigh lifts modified  Teaser III  Hip circles  Leg Pull Front  The leg pull-up  Side Bend  Rowing 1  The Boomerang  Push-ups  Rocking Swing  Twist I |
|  |  |  |

## Session C

| **BASIC**  **Session1-7** | **INTERMEDIATE**  **Session 8-21** | **ADVANCED**  **Session 22-36** |
| --- | --- | --- |
| **Exercises (15 repetitions)** | **Exercises (12 repetitions)** | **Exercises (10 repetitions)** |
| Preparation 1 to The Hundred  Roll Up modified  Preparation to Single Leg Circles  Single leg stretch  Double leg stretch modified  Obliques  Spine stretch forward modified  Saw  Obliques Roll Back  Preparation to  Shoulder Bridge  Sidekicks/up down  Sidekicks/small circles  Side leg lift/ lateral flexion  Preparation to Teaser  Preparation to Swimming  Preparation to Side Bend  PreparationtoPush-ups | Preparation 3 to The Hundred  Roll Up  Roll Over  Single Leg Circles  Single leg Stretch  Double leg stretch  Crisscross  Spine Stretch  Open Leg Rocker  Saw modified  Neck Pull  Shoulder Bridge  Preparation to Jack knife  Sidekicks/front back  Sidekicks/up down modified  Sidekicks/inner-thigh lifts  Teaser I  Preparation to Hip Circle  Preparation to Leg Pull Front  Preparation to The leg pull-up | The Hundred  Single straight leg stretch modified  Double straight leg stretch modified  Corkscrew modified  Scissors  Shoulder Bridge modified  Spine Twist  Jack knife  Sidekicks/front back modified  Sidekicks/up down modified  Sidekick/Grand Rond de Jambe  Teaser II  Hip circles  Leg Pull Front  The leg pull-up  Push-ups The  Rowing 1  Boomerang  Rocking  Side Bend |
|  |  |  |
